# Gene-Environment Interaction Research and Transgenic Mouse Models of Alzheimer's Disease

**DOI:** 10.4061/2010/859101

**Published:** 2010-10-05

**Authors:** L. Chouliaras, A. S. R. Sierksma, G. Kenis, J. Prickaerts, M. A. M. Lemmens, I. Brasnjevic, E. L. van Donkelaar, P. Martinez-Martinez, M. Losen, M. H. De Baets, N. Kholod, F. van Leeuwen, P. R. Hof, J. van Os, H. W. M. Steinbusch, D. L. A. van den Hove, B. P. F. Rutten

**Affiliations:** ^1^School for Mental Health and Neuroscience (MHeNS), Faculty of Health, Medicine and Life Sciences, European Graduate School of Neuroscience (EURON), Maastricht University Medical Centre, P.O. Box 616, 6200 MD Maastricht, The Netherlands; ^2^Department of Neuroscience, Mount Sinai School of Medicine, One Gustave L. Levy Place, New York, NY 10029, USA; ^3^Division of Psychological Medicine, Institute of Psychiatry, De Crespigny Park, London SE5 8AF, UK; ^4^Department of Psychiatry, Psychosomatics and Psychotherapy, University of Würzburg, 97080 Würzburg, Germany

## Abstract

The etiology of the sporadic form of Alzheimer's disease (AD) remains largely unknown. Recent evidence has suggested that gene-environment interactions (GxE) may play a crucial role in its development and progression. Whereas various susceptibility loci have been identified, like the apolipoprotein E4 allele, these cannot fully explain the increasing prevalence of AD observed with aging. In addition to such genetic risk factors, various environmental factors have been proposed to alter the risk of developing AD as well as to affect the rate of cognitive decline in AD patients. Nevertheless, aside from the independent effects of genetic and environmental risk factors, their synergistic participation in increasing the risk of developing AD has been sparsely investigated, even though evidence points towards such a direction. Advances in the genetic manipulation of mice, modeling various aspects of the AD pathology, have provided an excellent tool to dissect the effects of genes, environment, and their interactions. In this paper we present several environmental factors implicated in the etiology of AD that have been tested in transgenic animal models of the disease. The focus lies on the concept of GxE and its importance in a multifactorial disease like AD. Additionally, possible mediating mechanisms and future challenges are discussed.

## 1. Introduction

Alzheimer's disease (AD) is the most common form of dementia, characterized by an initial loss of short-term memory, followed by a progressive impairment in multiple cognitive domains. The estimated lifetime risk for developing AD is about 20% for women and 10% for men aged above 65 [1]. The pathology of AD is characterized by an accumulation of misfolded proteins, oxidative damage, and inflammatory changes ultimately resulting in region-specific loss of synaptic contacts and neuronal cell death [2]. Current biological theories on the etiology and pathology of AD posit central roles for age-related molecular and cellular aberrations that induce an imbalance in the production, cleavage, and clearance of amyloid-*β* (A*β*), hyperphosphorylation of the tau protein, and aberrant apolipoprotein E (APOE) function in the aging brain [1]. Several genetic risk factors have been linked with an increased risk of developing AD, such as mutations in the amyloid precursor protein (APP) and presenilin (PS) 1 and 2 for the familial cases of AD (FAD), as well as the APOE4 allele for the sporadic late-onset form of AD (LOAD). Several new genetic findings derived from powerful genome-wide association studies (GWAS; see below) have confirmed that AD is a polygenic disorder. The genes identified in these studies may enlighten unknown biological pathways involved in AD [3].

 Furthermore, various environmental exposures have been found to modify the risk of AD, such as diet and nutrition, physical exercise, exposure to metals, and brain trauma. Comorbidities, such as vascular disorders or depression, could also be of considerable importance, since these have also been suggested to contribute to the risk of AD. Recent evidence indicates that more attention should be paid to the role of the environment and its interactions with underlying genetic susceptibility in triggering disease-related phenotypes [4]. The gene-environment interaction (GxE) approach differs from the linear approach of either genetic or environmental effects by positing a causal role not only for either genes or environmental exposures in isolation, but for their synergistic participation in leading to a certain phenotype (here AD), where the effect of one is conditional for the other [5–7]. Where epidemiological studies on AD may reveal statistical evidence for GxE in the onset and course of AD, animal research can be instrumental in studying the underlying biological mechanisms. 

### 1.1. Objective

The objective of this review is to give an overview of the available transgenic mouse studies on AD, specifically addressing the concept of GxE. We start with a brief description of the various genetic and environmental risk factors of AD, and the different available transgenic mouse models of AD. The main part of the paper describes the effects of several environmental exposures on AD-related phenotypes. These sections begin with a brief description of the epidemiological evidence in AD (when available from meta-analyses) and continue with describing the findings from experimental animal studies in which the environmental factor was manipulated in AD transgenic mice and, when performed, in wild-type (WT) mice. Thereafter, we discuss the strengths and limitations of these studies, and we end with identifying future challenges and prospects.

## 2. Alzheimer's Disease

### 2.1. Genetics of AD

 Twin studies on AD have shown a heritability of 60%–80% and a concordance of 18% up to 83%, depending on for example, the population and age of the subjects investigated. Thus, both heritable and nonheritable factors play an important role in AD's age of onset, risk and etiology [8–11]. Several genetic risk factors have been linked to AD. Mutations in APP, PS1, and PS2 genes have consistently been associated with early-onset FAD. Also for LOAD several susceptibility loci have been linked with risk for AD, such as the gene encoding for the APOE4 allele or loci in the clusterin (CLU), phosphatidylinositol binding clathrin assembly protein (PICALM), complement receptor 1 (CR1), BIN1 (bridging integrator, amphiphysin) genes, a locus near the BLOC1S3 (biogenesis of lysosomal organelles complex1, subunit 3), and MARK4 (microtubule affinity-regulating kinase 4) genes [3, 12–14]. Other susceptibility loci have also been associated with AD (see [12, 15], http://www.alzgene.org/).

### 2.2. Environment and AD

Although a range of environmental exposures have been linked to AD, well-replicated and meta-analyses' evidence for the involvement of clear environmental factors in AD is sparse. Recent studies, however, have shown that dietary factors, such as exposure to a Mediterranean diet, fish and high omega-3 diets, cigarette smoking, head trauma, infections, systemic inflammation, and metal and pesticide exposure can significantly alter an individual's risk of developing AD. In addition, psychosocial factors such as education, social network, leisure activities and physical activity, chronic stress, and depression also seem to be connected to the risk of developing AD [16–18]. Somatic factors that are under the direct influence of environmental exposures, such as blood pressure, obesity, diabetes mellitus, cardio- and cerebrovascular diseases, and hyperlipidemia, have additionally been implicated in AD etiology [16, 18].

### 2.3. Gene-Environment Interactions and AD

The field of GxE research appears very promising for psychiatry and neuroscience, albeit still little investigated in AD [19]. The notion of potential existence of GxE in AD has substantial impact on the interpretation of reports on genetic and nongenetic contribution to this disorder. Reported contributions of environmental and genetic factors to disease risk can be misleading, since they represent the environmental exposure in relationship with the genetic susceptibility or resilience to it [6]. Thus, the advantage of the concept of GxE is that it includes the genetic control of sensitivity to the environment. Additionally, the genome-wide genetic findings identify associations that also include underlying GxE [6]. In fact, evidence for GxE in AD has recently started to accumulate. For example, an interaction between the APOE4 allele and cholesterol levels has been shown to increase the risk of AD [20, 21]. Significant statistical interactions were also found between moderate consumption of alcohol and the APOE4 genotype, as well as for smoking and the APOE4 genotype [22, 23]. Furthermore, an interaction with this risk genotype and social factors, such as cohabiting with a partner has been found; APOE4 carriers who lost their partner before midlife showed an increased risk of developing AD, compared to married or cohabiting people [24]. 

**Figure 1:**
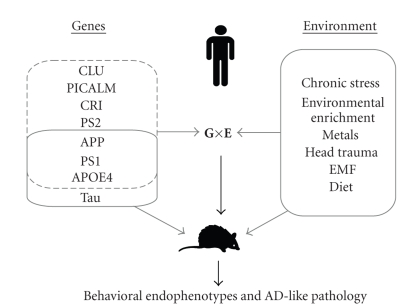
Research in Alzheimer's disease (AD) uses both clinical (human) and preclinical (mouse) methods to elucidate the underlying mechanisms of AD etiology. Epidemiological findings such as genetic and environmental risk factors can provide tools for investigating their effects on AD etiology separately in mouse models of AD. In this paper it is, however, postulated that AD research should move towards a gene-environment (GxE) interaction approach, so that the synergistic participation of genes and environment can be scrutinized. Genes in the dashed box represent those genes found to be implicated with Alzheimer's disease etiology in humans, while genes in the solid box resemble the genes that are currently used in mouse models of Alzheimer's disease. APOE4: Apolipoprotein *ε*4; APP: amyloid precursor protein; CLU: clusterin; EMF: electromagnetic field; PICALM: phosphatidylinositol-binding clathrin assembly protein; PS1: Presenilin 1; PS2: Presenilin 2.

These epidemiological studies indicate that it makes sense to focus future clinical AD studies on measuring both genes and environment and analyzing possible interactions, given that certain environmental factors may only affect a phenotype when the person is genetically endowed. A major drawback of epidemiological clinical studies is that they may indicate merely statistical interactions and thus cannot easily decipher the biological mechanisms that underlie the observed statistical interactions. Other major obstacles in clinical studies are the heterogeneity of the study population and co-occurrence of various environmental exposures in the same individuals. Experimental animal research has the advantage of enabling strict control of genetic and environmental variables. Recent advances in transgenesis allow altering specific genes in isolation, and in a time- and region-specific manner. As such, transgenic mice form a useful tool to study the effects of genetic and environmental variations and to identify the biological mechanisms that underlie the statistical GxE interactions observed in epidemiological studies (see Figure 1).

### 2.4. Transgenic Mouse Models of AD

Without the intention of giving a full overview of the available AD mouse models, some details on the types of transgenic mice that are discussed in the present paper can be found in Table 1. Information on the promoters used for the transgenic construct, and further details on genetic background are not further discussed here as these aspects lie outside the scope of this paper. 

It is noteworthy that the A*β* sequence of WT rodents has a three amino acid difference compared to humans, making it less likely to aggregate and deposit into amyloid plaques [25]. Therefore, to study A*β* aggregation and plaque formation in rodents it is necessary to manipulate them genetically [25]. Most transgenic mouse models focus on overexpressing human APP, PS1, tau, or APOE variants.

## 3. Chronic Stress

### 3.1. Human Studies of Chronic Stress

Chronic stress has been implicated in the etiology of AD. The likelihood of developing AD has been shown to increase by a factor 2.7 with the personality trait distress proneness [25, 48, 49]. Moreover, AD patients show elevated plasma cortisol levels [50, 51] with higher levels of plasma cortisol being associated with a more rapid disease progression and cognitive deterioration [51, 52]. 

Sustained elevated levels of glucocorticoids can cause volumetric and dendritic changes in the hippocampus of rats, mice, and tree shrews [53–56], decrease neurogenesis, and impair long-term potentiation [53, 57, 58]. It has, therefore, been proposed that alterations in HPA-axis functioning might also contribute to the etiology of AD [59–61]. 

Evidence from studies over the last 20 years indicates that major depression may serve as a risk factor for developing AD [62–69]. A lifetime history of depressive episodes doubles the chance of developing AD [70]. Interestingly, patients with major depression show a cerebrospinal fluid (CSF) profile of A*β*-species that resembles the profile seen in AD. They display decreased levels of A*β*42 and a decreased A*β*40 : A*β*42 ratio [71], which are considered putative biomarkers for AD [72]. In addition, the severity of depression correlated with binding of 2-(1-{6-[(2-{^18^F}Fluoroethyl)(methyl)amino]-2-naphthyl}ethylidine)malononitrile, also known as FDDNP, a tracer that binds to plaques and tangles, in the temporal lobe [73]. Moreover, more plaques and tangles in the hippocampus as well as a more rapid cognitive decline have been observed in AD patients with a lifetime history of major depression compared to patients without such history [74]. In contrast, others have suggested that major depression does not function as an independent risk factor for AD, but should merely be viewed as an AD prodrome [63, 75, 76].

### 3.2. Animal Studies of Chronic Stress

Several paradigms have been used to model the effects of chronic stress in mouse models of AD. The paradigms that have been applied most frequently are chronic isolation stress and chronic restraint or immobilization stress. Table 2 summarizes the current evidence for effects of stress exposure in transgenic mouse models of AD.

Chronic isolation stress by subjecting mice to either 3, 5, or 6 months of social isolation from weaning, has thus far only been used in the Tg2576 mouse model of AD [77–79]. This resulted in elevated levels of soluble A*β*40 and A*β*42 up to 59% and increased plaque deposition in the hippocampus and the neocortex [77, 78]. Moreover, this stress exposure paradigm caused a rise in basal plasma corticosterone levels, paralleled with an increased expression of the glucocorticoid receptor (GR) and corticotropin-releasing factor (CRF) receptor 1 [78]. In addition, impaired contextual memory and decreased cell proliferation in the hippocampal dentate gyrus was observed. Interestingly, the effects of isolation stress on memory deficits and cell proliferation in the dentate gyrus could be prevented by a 14-day treatment of fluoxetine [77].

Another widely used stress paradigm is restraint stress. Acute short-term restraint stress elevated intracerebral interstitial A*β* levels in Tg2576 mice [79] and stress-induced corticosterone release in APPswe mice [81]. Administering CRF or a CRF-antagonist indicated that the interstitial rise in A*β* depended on CRF levels [79]. Acute restraint stress furthermore resulted in a 175% increase in blood glucose levels in APPswe mice, suggesting a wide impact on metabolism [81].

Chronic restraint stress has so far been performed in 3 different mouse models of AD: APPV717I-C100, Tg2576, and PS1-L286V mice. Applying chronic restraint stress to APPV717I-C100 and Tg2576 mice generally resulted in an increased A*β* plaque load, increased A*β*40 and A*β*42 levels, increased tau phosphorylation and increased basal plasma corticosterone levels [82–84]. Chronic restraint stress applied to APPV717I-CT100 mice additionally induced cognitive impairment as measured for example by using cued food, that is, powdered chow mixed with a certain aroma, in the social transfer of food preference task [82]. Chronic restraint stress has also been associated with neuropathological alterations in AD mouse models. PS1-L286V mice exposed to chronic restraint stress displayed elevated numbers of degenerating neurons and a decreased number of proliferating cells in the hippocampus as compared to nonexposed mice [84]. Chronic restraint stress in APPV717I-CT100 mice caused elevated numbers of pyknotic cells in the hippocampus [82] and reduced dendritic arborization of cortical neurons in Tg2576 mice [83].

Another method to assess the effects of stress is by mimicking the physiological stress response by administering synthetic glucocorticoids, such as dexamethasone, for 7 days. Application of this approach in 3xTg mice resulted in elevated A*β*- and tau-immunoreactivity in the hippocampus, amygdala and neocortex and increased levels of insoluble A*β*40 and A*β*42 and total APP, *β*-site of APP cleaving enzyme (BACE1) and APP fragment C99 levels in brain homogenates [85].

## 4. Environmental Enrichment

### 4.1. Human Studies of Environmental Enrichment

A reduced risk for developing and a slower rate of cognitive decline have been observed in people having a greater purpose in life and higher levels of physical activity [86, 87].

### 4.2. Animal Studies of Environmental Enrichment

In the field of animal research, the environmental enrichment (EE) paradigm is frequently used to manipulate physical activity and social interactions. By introducing mates (social interaction) and/or toys (physical activity) into the cage of the rodent [88], this paradigm stimulates cognition as well as sensory and motor behavior with concomitant intracerebral cellular and molecular changes [89, 90]. To examine the effect of EE in AD several different paradigms have been imposed on various mouse models of AD. Table 3 summarizes the effects of EE in transgenic mouse models of AD.

#### 4.2.1. APP Mice

EE, in terms of housing multiple mice in a larger cage with platforms, running wheels, toys, and other novel habitats, for a period of 6 months improved cognition in a battery of tests such as Morris water maze (MWM), circular platform, platform recognition and radial arm water maze, despite signs of stable A*β* deposition in 16-month-old APPswe mice [91]. EE for 4 months in 5-month-old TgCRND8 mice did not significantly alter soluble levels of A*β* in the brain or the blood, but did enhance mRNA expression of angiogenic genes [92]. EE in this mouse model attenuated age-related reductions in cell proliferation, neurogenesis and synaptic plasticity [93], while the same paradigm in another laboratory elevated A*β* plaque load without compromising behavioral phenotypes such as feeding and drinking pattern, grooming, locomotion or cognition [94, 105]. In an attempt to disentangle the exact components of EE that influence phenotypes in APP mutant mice, Wolf et al. [96] exposed APP23 transgenic mice to either an enriched environment or unlimited access to a running wheel and compared both conditions with standard housing. EE had differential effects upon improving performance in the MWM as compared to the increased physical activity and standard housing groups, however, no differential effects on plaque load in the neocortex or hippocampus were found [95, 96]. Moreover, mice exposed to EE exhibited signs of increased hippocampal neurogenesis and neurotrophic support [95, 96].

#### 4.2.2. APP/PS1 Mice

When comparing social interaction and physical activity, differential effects of EE can be observed on learning and memory processes, A*β* plaque load and synaptophysin immunoreactivity of 9-month-old APP/PS1 transgenic mice [97]. EE in APPswe/PS1ΔE9 mice reduced cortical and hippocampal A*β* deposition with mice being more active in the running wheel showing an even more marked decrease in A*β* [98]. Furthermore, EE in PS1/PDAPP mice attenuated cognitive impairments [99]. 

Possibly in contrast with overt beneficial effects, 2-month-old female APPswe/PS1ΔE9 mice exposed to EE for several months displayed increased A*β* levels in the neocortex, and hippocampus [100]. After further backcrossing these mice to a C57Bl6 background strain in order to attain fewer genetic background differences, the same group demonstrated that EE in 2-month-old transgenic APPswe/PS1ΔE9 female mice, attenuated cognitive deficits [101], but still exhibited a 25% increase in A*β* deposits in cortical and hippocampal brain regions [101]. One could argue that enhanced secretion and deposition of toxic soluble A*β* species (scavenging the toxic species into packages away from intracellular and synaptic compartments) may be a mechanistic explanation for these findings.

#### 4.2.3. APOE Mice

EE in mice carrying the APOE3 allele improved learning and memory, as assessed with the T-maze test, while it had no effect in the ones carrying the E4 allele. The improved cognitive performance in APOE3 mice was associated with increased neocortical and hippocampal synaptophysin- and nerve growth factor-immunoreactivity, which was not observed in the APOE4 mice [102]. 

In conclusion, the majority of studies indicate that EE affects AD-related phenotypes in transgenic mouse models of AD pathology, mostly in a beneficial manner, particularly with regards to behavior. However, contradictory results have been reported which can possibly be explained by different experimental paradigms, age, sex, and genetic background of the mice used.

## 5. Metal Exposure

### 5.1. Lead

Lead exposure has been proposed as a risk factor for AD by some authors [106, 107] while others have argued against it [108]. No studies to date have performed lead exposure experiments in mouse models of AD although other animal work has indicated that lead exposure early in life may contribute to the onset of AD-related pathology later in life [109, 110].

### 5.2. Aluminum

While it has been proposed that occupational aluminum exposure is not a significant risk factor for AD [108], prolonged exposure to aluminum in drinking water is significantly associated with an increased risk of developing AD in a dose-dependent manner with the relative risks varying from 1.00 to 2.14 (for review see [111]). These findings should be regarded with some caution as aluminum concentrations varied highly between the different studies and many variables (such as interaction with other chemical constituents in the drinking water as well as alternative sources of aluminum, for example through antacid use or dietary intake) have often been overlooked in these studies [111]. 

Products made of baking-powder often contain high levels of aluminum, and it has been observed that AD patients were more frequently exposed to ingestion of foods containing baking-powder (retrospectively investigated) than age-matched controls [112]. Other studies suggested that AD patients have significantly enhanced gastrointestinal absorption of aluminum (up to 1.64 times higher) compared to age-matched controls, and indicate that differential gastrointestinal function may lead to a systemic rise of aluminum [113, 114]. 

Praticò and colleagues [115] reported that Tg2576 mice exposed chronically to dietary aluminum displayed increased A*β*40 and A*β*42 levels, plaque deposition, and markers of oxidative stress in the hippocampus and neocortex compared to non-exposed Tg2576 mice. Others authors, however, were not able to replicate these findings. They reported that chronic aluminum treatment in Tg2576 mice did not affect A*β* load in the cerebral cortex or oxidative stress reactions in the hippocampus, nor impair spatial cognition, as measured by the MWM [115–117]. Aluminum treatment did raise the levels of aluminum and other metals in the hippocampus, neocortex and cerebellum, but no major differential effects could be found between Tg2576 and WT mice [116, 118]. The differential effect of aluminum exposure in these mice could possibly be explained by higher concentrations of aluminum in the chow, differential ages at the start of the experiment and a shorter duration of exposure.

### 5.3. Iron, Zinc, and Copper

The endogenous biometals iron, zinc and copper have often been implicated in AD, as they are present in and around amyloid deposits in the AD brain and their presence can promote aggregation of A*β* [119–121]. 

Although no report has confirmed a direct link between iron exposure and the risk of developing AD, Dwyer and colleagues do propose a ferrocentric model of AD [122]. Higher levels of ferritin iron in the basal ganglia have been considered a risk factor for AD [123, 124]. AD patients show elevated levels of iron in the hippocampus [125], and this metal seems to concentrate in the core and rims of plaques in the amygdala [126].

Rodent research has indicated that gestational or early developmental iron deficiency can alter the expression of the APP and CLU genes implicated in synaptic plasticity, dendritic outgrowth, and AD pathogenesis [127–129]. Neonatal administration of iron for 3 days to APPswe/PS1ΔE9 mice was found not to alter A*β* deposition in the hippocampus and temporal cortex at 6 months of age but did cause changes in lipid composition, decreased steady-state levels of oxidative damage markers, and increased astrocyte levels in the temporal cortex [130]. 

Zinc seems to play a double role in AD etiology. Low levels of zinc have been reported to be protective against A*β* formation [131] and metalloproteases, such as neprilysin and insulin-degrading enzyme (IDE), that degrade A*β* are zinc dependent [132]. It has also been found that high levels of zinc elevate A*β* toxicity [131, 133] and promote total A*β* aggregation [121]. AD patients displaying higher levels of zinc in hippocampus and amygdala [125, 126] exhibited normal zinc serum levels, but significantly lower zinc levels in CSF compared to matched controls [134]. This may be explained by the binding of zinc to A*β* in the brain parenchyma [135]. However, to our knowledge, no reports have been published on putative associations between zinc exposure and risk of AD in the human population. Nonetheless, several studies have investigated the effects of altered zinc intake in AD mouse models.

Stoltenberg et al. [136] reported that lowering zinc by a 3-month dietary deficiency increased the plaque load in APPswe/PS1ΔE9 mice by 25%, without changing zinc ion distribution, zinc transporter mRNA expression levels nor inducing oxidative stress [136]. Alternatively, administrating zinc to TgCRND8 and Tg2576 mice through the drinking water for a period of 5 and 9 months, respectively, lowered the amyloid plaque burden in the hilar and molecular region of the dentate gyrus, while impairing spatial memory in MWM [137]. Concurrently, long-term administration of high zinc concentrations in TgC100 mice did not significantly affect soluble A*β* levels or levels of glial fibrillary acidic protein (GFAP), superoxidase dismutase 1, APP, *β*-secretase-cleaved carboxyl-terminal fragment, or neurofilament 200, a marker for neuronal damage [138]. Interestingly, a genetic reduction of zinc in the brain of Tg2576 mice, by crossing these mice with a zinc transporter 3 deficient mouse (ZnT3^−/−^), significantly reduced the plaque load in the hippocampus and neocortex while increasing the ratio between soluble versus insoluble A*β* [139]. 

AD patients have been shown to display reduced copper levels in the amygdala and hippocampus, while copper levels are specifically elevated in amyloid plaques [125, 126]. Copper intake in AD patients decreases the reduction of A*β*42 in CSF most typically seen as the disease progresses, but does not ameliorate cognitive performance [140]. Adding copper to drinking water of cholesterol-fed rabbits causes accumulation of A*β* and the formation of plaque-like structures [141]. 

Exposing AD mouse models to chronic upregulation of copper has yielded conflicting results. Chronic copper administration to APP715SL mice did not alter copper, zinc, iron, A*β* nor APP levels in the brain [142]. Long-term administration of high levels of copper resulted in a 18% decrease in soluble A*β*40 and increased zinc levels in the brain without changing GFAP, SOD1, APP, C100, or NF200 levels to TgC100 mice. Yet, copper exposure in 3xTg mice led to elevated steady-state levels of APP, and C99 as well as to increased A*β* production and tau phosphorylation in the brain [143]. Interestingly, copper, APP and A*β* seem to be closely connected; Tg2576 mice displayed an overall reduction of copper in the brain whereas the ablation of APP and amyloid precursor-like protein 2 increased overall central copper levels [144, 145]. 

In summary, both human and rodent research on the exact contributing roles of metal exposures in interaction with AD risk genes APP and PS1 remain largely inconclusive. For an overview of metal exposure in the transgenic animal models of AD listed above, see Table 4.

## 6. Traumatic Brain Injury

### 6.1. Human Studies of Traumatic Brain Injury

Traumatic brain injury (TBI) has repeatedly been identified as a risk factor for AD. It has been suggested that TBI accelerates the onset of AD and that the severity of the injury increases the risk of AD [147]. AD-like pathology has been observed after acute brain trauma, even in brains of young individuals. A polymorphism in the promoter of the gene that encodes neprilysin, causing a greater length in GT repeats, has been associated with the acute development of plaques following TBI [148]. In addition, carriers of the APOE4 genotype have been associated with poorer outcome after TBI [147].

### 6.2. Animal Studies of Traumatic Brain Injury

For an overview of TBI in mouse models of AD, see Table 5. After corticol contusion, 10- to 16-month-old PDAPP mice did not show significant differences in behavior or A*β* neuropathology following TBI, as compared to WT controls that underwent the same procedure of experimental brain injury [149]. Inducing TBI in the PDAPP mouse model at 4 months of age, accelerated memory loss as assessed with the MWM test. TBI also resulted in hippocampal neuronal loss one week after injury, which was associated with an increase in hippocampal A*β*40 and A*β*42 [150]. Furthermore, TBI resulted in long-term effects at 2, 5, and 8 months after TBI: a significant reduction in A*β* plaque load was found which was accompanied with more pronounced hippocampal atrophy. TBI induction in APP^NLh/NLh^ mice caused a twofold increase in soluble hippocampal A*β* levels at 3 and 7 days after TBI. Additionally, post-TBI administration of caspase-3 inhibitors and the hypolipemic simvastatin were able to attenuate impaired hippocampal synaptic function, microglial activation and MWM performance after TBI induction in APP^NLh/NLh^ mice [151, 152]. TBI induced by cortical impact provoked gene expression changes in 22-month-old APPswe mice compared to WT mice. Expression changes were detected in genes involved in various biological pathways such as immune response, cell cycle and cell death, cellular development, tissue development and connective tissue function and development, cellular movement, and hematological systems [153]. 

 Single and repetitive mild TBI, using a cortical impact device, in 9-month-old Tg2576 mice as compared to sham treated transgenic and WT, resulted in significant cognitive dysfunction (measured with MWM) without affecting motor performance 16 weeks after TBI. However, only repetitive TBI caused increased A*β* burden in the hippocampus and neocortex with a parallel increase in isoprostane, an indicator for increased oxidative stress [154]. TBI in 10-month-old transgenic mice overexpressing either human APOE3 or 4, was associated with differential gene expression, particularly in genes related to oxidative stress, with an increased expression of antioxidant genes in the APOE3 mice as compared to the APOE4 [155]. 

Thus, accumulating evidence indicates that TBI interacts with AD-related genes.

## 7. Electromagnetic Field Exposure

Occupational exposure to electromagnetic field (EMF) has been proposed as a risk factor of AD. In particular, extremely low-frequency exposure has been implicated to increase the odds to develop AD up to 2.03 (as reviewed in [155]). Strikingly, Arendash et al. [27] demonstrated that long-term high-frequency exposure to EMF (i.e., similar to that generated by cell-phone use) was beneficial to A*β*PPswe mice (see Table 4). EMF exposure in young adult A*β*PPsw mice prevented the age-related genotype-specific cognitive impairment, while EMF in aged A*β*PPsw mice was also able to reverse cognitive impairment in these animals. Chronic EMF exposure furthermore influenced A*β* aggregation in the brain, with higher levels of soluble A*β* and less A*β* plaques in the hippocampus and entorhinal cortex of A*β*PPsw mice. EMF exposure has been proposed to contribute to a decrease in A*β* aggregation, via altering levels of transthyretin [158]. Transthyretin is known to sequester A*β* in CSF, thereby hindering its aggregation into amyloid plaques [158]. Interestingly, AD patients show a significant decrease in CSF transthyretin levels [159] while decreased transthyretin levels have also been found in blood serum of long-term wireless phone users [158]. Thus, effects of EMF are quite puzzling while the association with AD remains to be firmly established.

## 8. Effects of Diet and Nutritional Factors

### 8.1. Mediterranean Diet

Various dietary and nutritional factors seem to be protective or detrimental in the development and course of AD. One of the most prominent is the Mediterranean type of diet which has been linked to reduced risk of developing AD and showing a dose-response effect (high adherence to Mediterranean diet, OR: 0.76; moderate adherence, OR: 0.47) [160–162]. 

A typical Mediterranean diet is characterized by higher consumption of vegetables, fruits, cereals, fish, and olive oil and associated with a general higher consumption of unsaturated fatty acids and lower consumption of saturated fatty acids, usually accompanied by mild or moderate alcohol intake (preferably red wine taken with meals) [160]. The exact factors and mechanisms by which the Mediterranean diet is protective remains to be elucidated, although it has been speculated that this diet can attenuate the detrimental effects of oxidative stress and inflammation [161].

### 8.2. Western Diet and Obesity

Obesity during mid-life is associated with an increased risk for AD, with an OR of 2.4, additively increasing up to 6.2 when combined with high total cholesterol levels and high systolic blood pressure [163]. Higher intake of calories and fat have been associated with increased risk for developing AD, particularly in APOE4 carriers, with a hazard ratio of 2.3 [164]. Western, high-fat and low carbohydrate diet for 4 months in 1-month-old Tg2576 mice, increased levels of soluble A*β* in brain homogenates, while the treatment did not have any effect on plaque load [165]. Further, insulin resistance induced by 5 months of high-fat diet, in 9-month-old Tg2576 mice, was associated with to a twofold increase of A*β*40 and A*β*42 peptide content in the hippocampus and a twofold increase in plaque burden in the neocortex, with a concomitant acceleration of cognitive decline as measured by the MWM. In addition, *γ*-secretase activity was increased, while the expression of IDE was decreased by this diet [166]. APP/PS1 KI mice exposed to Western, high-fat diet showed increased oxidative stress markers as measured in brain homogenates of 2-month-old mice when compared to nontransgenic controls, but A*β* levels were not altered [167]. In another study, Western diet increased A*β* deposition in the hippocampus of the APPswe/PS1ΔE9 transgenic mice at 18 months of age, after a period of 12 months on a high-fat diet [168]. In the 3xTg mouse model of AD, a high fat diet starting at the age of 4 months for a total period of 13 months, induced similar effects in the frontal cortex [169].

### 8.3. Cholesterol

As the generation, deposition, and clearance of A*β* is regulated by cholesterol, many studies have specifically focused on the implication of lipids, cholesterol metabolism, related vascular disease, APOE genotype, and their interrelationships on the development of AD [170–172]. The precise mechanisms underlying cholesterol and APOE4 need further investigation, as it is not clear whether cholesterol and the APOE4 genotype act as independent factors or interact with one another or whether the effect of APOE4 is partially mediated by high cholesterol levels [171–174]. Also, hypercholesterolemia in 3-month-old APP_swe_/PS1_M146L_ mice has been shown to accelerate A*β* accumulation while drug-induced hypocholesterolemia reduced the amyloid pathology [175, 176].

### 8.4. Docosahexaenoic (DHA)

Studies in mouse models of AD amyloidosis, such as Tg2576, APPswe/PS1ΔE9, and 3xTg, have shown that a diet rich in the omega-3 fatty acid DHA reduces A*β* accumulation and somatodendritic tau accumulation, improves cognition, and induces cerebral hemodynamic changes [168, 177–180]. Such findings are in line with evidence from epidemiological studies showing a protective effect of diets rich in omega-3 fatty acids [181–183]. More specifically, DHA-enriched diet was shown to increase relative cerebral blood volume with a concomitant improvement in spatial memory and reduction of A*β* load in APP_swe_/  PS1_M146L_ mice [184]. Exposing APPswe/PS1ΔE9 mice to a diet high in omega-3 fatty acids, however, neither improved cognition in APPswe/PS1ΔE9 mice nor reduced hippocampal A*β*, but increased omega-3 fatty acid levels in their brain [185]. Interestingly, high levels of omega-6 were linked to cognitive impairment [185].

### 8.5. Vitamins

Dietary deficiency of B6, B12, and folate for 7 months increased A*β* levels in the brains of 15-month-old Tg2576 mice, without altering APP, BACE-1, A disintegrin and metallopeptidase 10 (ADAM-10), nicastrin, IDE, APOE, or neprilysin [186]. Additionally, the same pattern of dietary vitamin B deficiency led to increased expression of PS1 via DNA demethylation of the promoter region of the encoding gene in brain homogenates of TgCRND8 mice [187]. In the brains of mice of the same animal model, vitamin B deficiency increased the levels of glycogen synthase kinase 3*β* (GSK3*β*) and reduced the activity of protein phosphatase 2A, which are both involved in the hyperphosphorylation of tau [188]. Furthermore, folic acid deficiency for 3 months in APPswe mice did not affect the A*β* plaque load, but induced neuron loss in the CA3 region of the hippocampus and enhanced hippocampal DNA damage, as compared to controls [189]. Besides B6 and B12, deficiency of B1, also called thiamine, exacerbated A*β* pathology via an upregulation of BACE1 in brains of Tg19959 mice [190]. 

Furthermore, dietary supplementation with the coenzyme Q10 for 2 months delayed hippocampal atrophy in 22-month-old APP_swe_/PS1_Leu235Pro_ mice as compared to vehicle treated controls [36, 37], with concurrent reduction in plaque load [36, 37]. 

Deficiency of vitamin A has been implicated in A*β* accumulation, loss of long-term potentiation and memory impairment, while administration of its active metabolite retinoic acid for a duration of 2 months was able to rescue these deficits in the frontal cortex and hippocampus of 7-month-old APPswe/PS1ΔE9 transgenic mice [191].

### 8.6. Caffeine and Green Tea

Besides the various nutritional factors, other lifestyle habits have also been associated with AD. Longitudinal studies have shown that coffee and tea drinking are associated with decreased risk for cognitive decline, dementia and AD in various population samples [192, 193]. Another study showed a protective effect of caffeine only in women, with a relative risk of 0.49 [194], but a meta-analysis estimated an overall protective effect against dementia, with a relative risk of 0.84, though pointing out the large heterogeneity in the methods of the various epidemiological studies [195].

Acute and long-term caffeine consumption was recently shown to delay cognitive decline and lower A*β* pathology in the hippocampus of 15-month-old APPswe and APP_swe_/  PS1_M146L_ mice, by suppressing *β*- and *γ*-secretase levels [196, 197]. Furthermore, oral or intraperitoneal administration of epigallocatechin-3 gallate, which is derived from green tea, for 2 or 6 months, exerted beneficial effects in APPswe transgenic mice, at the age of 14 months. The beneficial effects consisted of a reduction in A*β* pathology in the neocortex and hippocampus, with a parallel improvement of working memory [198–200]. Furthermore, administration of the citrus-derived flavonoid luteolin and its analogue diosmin for a total of 30 days, significantly reduced A*β* pathology in the hippocampus and neocortex of 9-month-old Tg2576 mice. This effect was mediated via an inhibition of GSK3*β*, which increased PS1 phosphorylation [201].

### 8.7. Wine

Moderate red wine consumption has been shown to be beneficial. Cabernet sauvignon administration for 7 months in 4-month-old Tg2576 mice attenuated the cognitive impairment that is observed in these mice, in terms of spatial memory, when compared to ethanol-consuming and tap water Tg2576 controls. Cabernet sauvignon consumption decreased cortical and hippocampal A*β* plaque load in these mice, by promoting nonamyloidogenic processing in the direction of *α*-secretase cleavage [202]. Further, in vitro studies in hippocampal neuron cultures derived from Tg2576 mice, showed that the polyphenol extracts from the Cabernet sauvignon grapes increased the levels of *α*-secretase, which promotes the nonamyloidogenic cleavage of APP that reduced the levels of A*β* peptides [202]. Furthermore, consumption of the muscadine wine was proven to attenuate A*β* pathology in brains of 14-month-old Tg2576 mice, following a 10-month wine treatment, with a different mechanism of action. In this case, muscadine consumption reduced the aggregation of A*β*, with a parallel improvement in spatial memory [203]. The differential effect of the two types of wine was attributed to their distinct composition in polyphenolic compounds, which have a differential effect on APP processing [203].

### 8.8. Nicotine

Smoking in humans has been linked with increased risk for AD [204–206], while nicotine administration for 6 months to transgenic mice carrying the APPswe mutation reduced the levels of insoluble A*β* in various brain regions of 15-month-old mice [207, 208]. Nevertheless, nicotine administration for 5 months exacerbated hippocampal tau pathology in 6-month-old 3xTg mice, increasing tau hyperphosphorylation and aggregation, in combination with an earlier onset of these tau-related changes compared to controls [209].

### 8.9. Caloric Restriction

Caloric restriction in animals has been found to prolong mean and maximum life span, reduce body fat, attenuate age-related molecular changes, and slow the decline associated with aging in various species [210]. In nonhuman primates, for example, caloric restriction prolongs lifespan and delays the onset of diseases, such as cardiovascular diseases, diabetes, and brain atrophy [211]. Restricting caloric intake by 40% for 6 weeks in APPswe and 14 weeks in APP_swe_/  PS1_M146L_ mice has been shown to reduce the number and size of amyloid plaques by 40% and 55%, respectively, while also reducing the plaque-related astrocyte activation. These effects were observed in the neocortex and hippocampus [210]. Similar effects have been observed in the hippocampus of the 3xTg model after 14 months of caloric restriction [212].

### 8.10. Others

In line with studies showing a beneficial effect of nonsteroidal antiinflammatory agents on AD pathology in transgenic models of AD [213], the phytogenic curcumin (a major dietary component in India) administered for 6 months reduced levels of soluble and insoluble A*β*, plaque load, oxidative stress and inflammatory response in various brain regions of 16-month-old transgenic mice carrying the APPswe mutation [214]. Additionally, blueberry supplementation and *Gingko biloba *extract treatment have been found to improve memory deficits in APP/PS1 and Tg2576 mice without affecting amyloid plaque load [215, 216]. An overview of all nutritional and dietary factors influencing animal models of AD is given in Table 6.

## 9. Strengths, Limitations, and Future Challenges of G×E Research in AD

This paper provides an overview of experimental mouse data on environmental exposures known to be associated with AD. In general, it may be concluded that many studies have shown effects of environmental manipulations on a wide variety of phenotypes in transgenic mouse models of AD. 

It is challenging to evaluate the exact role of GxE in the field of preclinical AD research, due to several limitations. Numerous mouse models have been used on different genetic backgrounds and at multiple ages, applying various protocols of experimental exposures. This high level of variability makes it difficult to draw firm and general conclusions on any of the discussed exposures. In addition, the read-out parameters differed per group, with some focusing on A*β* pathology, synaptic integrity, or oxidative stress and others emphasizing behavioral effects. Another limitation is that most animal models used have focused on fAD mutations, while a GxE be more applicable in sporadic forms of AD. 

The experimental design of most of the available studies often consisted of testing the effects of environmental exposures only in transgenic mice, without full comparison of the effects of those environmental exposures in wild type animals. In such an experimental setup, one can merely study disease acceleration or progression as a result of environmental exposure. Additionally, some environmental factors may have a differential effect in the initiation of the disease from their effect on disease progression. Evidently, this type of research can still provide us with insights on underlying biological mechanisms, but it cannot disentangle the synergistic participation of genes and environment in the induction of an AD phenotype. 

In a clinical setting, however, GxE in AD etiology is particularly complex to decipher with standard epidemiological designs, particularly because the time-window between environmental exposures during life and the clinical phenotype of AD is very long. The high variability in environmental exposure across the life span also makes it challenging to capture this interaction. 

Most AD research over the past decades has been A*β*-centered, yet recent clinical trials based on the amyloid cascade hypothesis have yielded controversial and sometimes disappointing results [219]. Similarly, the majority of animal models of AD have also grossly been focused on A*β*-enhancing mutations. Furthermore, outcome measures on the effects on environmental factors are often expressed in terms of soluble and insoluble A*β* and plaque load alterations. Because the extent of amyloid burden does not correlate well with AD symptomatology, one could argue that it would be advantageous to design future studies in a multidisciplinary manner encompassing a wide range of outcome measures such as behavioural phenotypes, biochemical, molecular, as well as neuropathological alterations and use similar outcome measures in the various studies conducted by different research groups so that results are more comparable. 

 Possible biological mechanisms that mediate the effects of environmental exposures and that could be the focus of further translational AD research are, among others, inflammation, oxidative stress, protein misfolding, glucose metabolism, and epigenetics [19, 220–223]. The AD brain shows ample signs of ongoing inflammatory processes, such as the presence of proinflammatory cytokines and activated microglia surrounding amyloid plaques [224]. A large body of evidence has pointed towards a role of oxidative stress and oxidative damage in brain regions that are affected by AD [220]. In addition, changes in the epigenome have been implicated in the pathophysiology of AD that can be triggered by various environmental factors [19], while the exact role of misframed proteins, such as ubiquitin+1 that have been found to accumulate in AD, remains also to be fully elucidated [223, 225].

## 10. Concluding Remarks

It appears likely that a large part of, at least, sporadic cases can be connected with GxE, with the field being challenged to identify the most relevant GxE by for example conducting prospective clinical studies of subjects that will develop AD. Exposure to environmental risk factors during early life has been linked to other complex psychiatric phenotypes and disorders. For example, prenatal maternal and early life stress can be viewed as a risk factor for developing major depression and schizophrenia [226–228]. Likewise, environmental exposures during early life may well impact on the risk of developing AD. For example, neonatal exposure to metal lead has been proposed as an early environmental trigger for AD-related pathology in rodents and macaque monkeys [109]. Findings like these have spurred researchers to formulate the “Latent Early Associated Regulation” (LEARn) theory of AD pathogenesis proposing that indeed early environmental exposures can change gene expression for long time periods and can induce pathology that only becomes apparent later in life, after subsequent trigger(s) [109, 229]. 

While numerous challenges lie ahead, it can be argued that it is timely to move attention of epidemiological as well as experimental animal research in the field of AD towards the synergistic approach of GxE research. In a preclinical setting, one could envision focusing more on the use of recently identified genetic variants of the newly found GWAS genes. Second, current studies can benefit from the further technological advances in transgenesis that enable time- and region-specific expression of transgenes, thereby allowing for the investigation of GxE during specific time windows in development and aging, as well as in specific brain regions. Third, it may be valuable to scrutinize the roles of genetic and environmental risk factors (and their interactions) of diseases that have been associated with the onset of AD, such as cardiovascular disease and diabetes mellitus type 2. The underlying mechanisms of these disorders, which likely involve GxE, could shed a new light on the etiology of AD. 

To conclude, moving towards a GxE approach in both clinical and experimental animal studies seems promising in further elucidating the multifactorial etiology of AD, and in identifying modifiable factors that are of particular relevance for subgroups of AD patients. The further use and development of animal models combining genetic and environmental manipulations will be a driving force in elucidating the exact biological underpinnings of this detrimental disorder.

## Figures and Tables

**Table 1:** Transgenic mouse models of Alzheimer's disease with reported environmental effects.

Name	Mutation	Background	Effect	Ref.
3xTg	Injection of APPswe and tauP301L transgenes in PS1M146V knock-in mice	129/C57BL6	Intraneuronal A*β* at 3 months, extracellular at 6, hippocampal hyperphosphorylated tau pathology at 12 months, synaptic dysfunction	[26]

A*β* PPswe	Carrying the mutant A*β*PPK670N, M671L gene	Mixed background of 56.25% C57, 12.5% B6, 18.75% SJL, and 12.5% Swiss-Webster	Amyloid deposition and cognitive decline starting at the age of 8 months	[27]

APOE3, APOE4	APOE knockout mice carry an inactivated APOE endogenous gene disrupted by gene targeting in embryonic stem cells. Human APOE genomic DNA fragments injected in single cell emryos fertilized by APOE knockout mice	APOE knockout and C57BL6	Expression of human APOE in the brain, high cholesterol levels	[28]

APP23	cDNA of human APP with the Swedish double mutation at positions 670/671 combined with the V717I mutation, inserted to the blunt ended XhoI site of the expression cassette containing the murine Thy 1.2 gene	C57BL6	A*β* deposition in the neocortex and hippocampus at the age of 6 months	[29]

APP715SL	Swedish (KM670/671NL) and London (V717I) mutation under control of Thy1 promotor	CBA/C57BL6	Amyloid plaque deposits at 6 months of age	[30]

APP^NLh/NLh^	Human A*β* coding sequence knocked-in to the endogenous APP gene, combined with the Swedish (K670N/M671L) mutation	129/Sv	No A*β* depositions, but a 9-fold increase in human A*β* production compared to normal human A*β* levels	[31]

APP/PS1 KI	Double knock-in mouse: APP^NLh/NLh^ crossed with PS1 P264L knock-in, using Cre-lox knock-in technology and endogenous promoters	CD-1/129	Increase of A*β*42 levels, amyloid deposition and reactive gliosis by 4 months of age.	[32, 33]

APPswe/ind	Expressing human APP with Swedish mutation (K670N/M671L) and the V717I Indiana mutation under the PDGF promoter (J20 line)	C57BL6 × DBA/2	Increased A*β* production and A*β* deposition at 5–7 months of age, decrease in synaptophysin immunoreactivity at 2–4 months of age	[34]

APPswe/PS1ΔE9	Cross of APPswe and PS1ΔE9 (expressing human PS1 carrying the exon 9 deleted variant)	C57BL6J	Amyloid plaque deposition, cholinergic marker decrease, memory deficits at 6 months of age	[35]

APPswe/PS1Leu235Pro	APP swedish mutation crossed with mutant human PS1 Leu235Pro	C3H/HeJ/C57BL/A2G	—	[36, 37]

APPswe/PS1M146L	Tg2576 combined with PS1 (M146L) mutation (under PDGF promoter)	C57/B6/SJL/Swiss Webster	Compared to Tg2576, 41% increase in A*β*42 which precedes fibrillar A*β* deposits in cerebral cortex and hippocampus. Reduced spontaneous alternation performance in the Y-maze.	[38]

APPV717I-C100	Expressing the C-terminal 100 amino acid of human APP with 717 London mutation	C57BL6	Intracellular accumulation of soluble A*β*	[39]
APP-YAC	The entire human APP gene inserted to the yeast artificial chromosome (YAC) B142F9, introduced to embryonic stem cell by lipofection	C57BL6	Significant human APP expression in the cerebral cortex	[40]

PDAPP	Indiana mutation (V717F) with portions of APP introns 6–8, driven by the PDGF promoter		Extracellular A*β* deposits in the hippocampus from the age of 6 months and neocortex from 8 months of age	[41]

PS1-L286V	Overexpressing human PS1 with L286 mutation under the control of human PDGF-*β* promoter	FVB/N	A*β*42 intracellular deposits at 13 months of age	[42]

TASTPM	Carrying human APPswe and PS1 M146V mutations	C57BL63H	Cerebral A*β* deposition and cognitive deficits at 6 months of age	[43]

Tg19959	TgCRND8 mice plus M146L + L286V PS1 transgene in the hamster PrP gene promoter	C57/C3H/129SvEv/ Tac/FVB	Amyloid deposits at 1 month of age	[44]

Tg2576	Human APPswe (double K670N, M671L) inserted to hamster prion protein promoter (PrP) (is also known and referred to in the text as APPswe)	C57BL6	5-fold increase in A*β*40 and 14 fold increase in A*β*42, behavioral deficits, amyloid plaques at 9 months of age	[45]

TgC100	Expressing the C-terminal 100 amino acid of human APP (with or without 717 London mutation)	C57BL6	Intracellular accumulation of soluble A*β*	[39]

TgCRND8	Swedish and Indiana (V717F) APP mutations	C57/C3H/129SvEv/ Tac/FVB	Plaques at 3 months of age, increased A*β*42/40 ratio	[46]

TgV337M	V337M longest tau, cDNA inserted to the PDGF*β*-chain expression vector	B6SJL	Hyperphosphorylated tau aggregates in the hippocampus, neurodegeneration, reduced hippocampal neural activity and behavioral abnormality	[47]

**Table 2:** Effects of stress exposure in transgenic mouse models of Alzheimer's disease.

Mouse model	Exposure	Duration of the experiment	Age at the start	Effects on the brain	Effects on behavior	Reference
Tg2576	Chronic isolation stress	3 months	From weaning	↑ soluble A*β*40 (38%) and A*β*42 (59%) in hippocampus, no change in A*β*40 : A*β*42 ratio, no difference in APP, *α*- or *β*-CTF levels, no changes in IDE, NEP (neprilepsyn) or APOE levels	Not measured	[79]

Tg2576	Chronic isolation stress	5 months	From weaning	↑ A*β* plaques ↓ proliferation in DG	↓ contextual memory at 6 months	[77]

Tg2576	Chronic isolation stress	6 months	From weaning	↑ A*β*40 + A*β*42 levels and plaque deposition in neocortex and hippocampus ↑ expression of GR and CRFR1 in neocortex and hippocampus ↑ basal corticosterone in plasma	Not measured	[78]

TASTPM	Repeated novel cage exposure (1 h/day, 4x/week)	5 weeks	4 months	No changes in basal corticosterone levels ↓ soluble A*β*40 levels in the frontal cortex + hippocampus ↓ insoluble A*β*42 levels in frontal cortex + hippocampus, no difference in endocannabinoid levels in frontal cortex and hippocampus	No difference in locomotion, nor in anxiety levels contextual memory ↑	[80]

APPswe	Acute restraint stress (for 4 h)	4 hours	19 months	↑ 175% in blood glucose levels, dropping to below basal values 2 hours after restraint ↑ in stress-induced corticosterone release	Not measured	[81]

Tg2576	Acute restraint stress (for 3 h)	3 hours	3-4 months	↑ interstitial fluid A*β*, no difference in APP or *β*-CTF levels ↓*α*-CTF levels, no changes in IDE, NEP (neprilysin) or APOE levels	Not measured	[79]

APPV717I-CT100	Chronic immobilization stress (6 h/day, 4x/week)	8 months	3 months	↑ A*β* plaques in hippocampus, entorhinal + piriform cortex ↑ APP-CTFs ↑ pyknotic cells in hippocampus + entorhinal cortex ↑ phospho-tau in CA3 + entorhinal cortex ↑ Corticosterone in plasma	↑ cognitive impairment	[82]

Tg2576	Chronic immobilization stress (6 h/day, 4x/week)	6 months	3 months	Not measured	↓ cued food preference	[82]

Tg2576	Chronic restraint stress (2 h/day)	16 days	14 months	↑ A*β* plaques in hippocampus, PFC, cingulate, motor, parietal and piriform cortex ↑ A*β*40 + A*β*42 in cortical homogenates ↑ immunoreactive astrocytes near plaques ↑ phospho-tau ↓ dendritic arborization of cortical neurons ↓ MMP-2 (A*β*-degrading enzyme) ↑ basal corticosterone levels	Not measured	[83]
PS1-L286V	Chronic restraint stress (6 h/day)	3 or 15 weeks	7 weeks	*3 weeks exposure * ↓ body weight ↑ adrenal gland weight ↑ corticosterone levels in plasma ↑ number of degenerating neurons in DG, CA3, and retrosplenial cortex, no effect on number of granule neuron precursors (Pax6) or proliferating cells (Ki67) in DG and/or SGZ ↓ BrdU-positive cells ↑ DCX-positive neuronal progenitor cells *15 weeks exposure * ↓ body weight ↑ adrenal gland weight ↑ number of degenerating neurons in DG, CA3 and retrosplenial cortex, no effect on # of granule neuron precursors or proliferating cells in DG and/or SGZ	Not measured	[84]

3xTg	Dexamethasone administration (1 or 5 mg/kg) i.p.	7 days	4 months	↑ A*β* in hippocampus, neocortex, amygdala ↑ tau in dendrites and axons in hippocampus, neocortex, amygdala ↑ insoluble A*β*40 and A*β*42 ↑ total APP, BACE1, C99 levels ↑ basal corticosterone levels from 9 months on	Not measured	[85]

**Table 3:** Environmental enrichment in transgenic transgenic mouse models of Alzheimer's disease.

Mouse model	Exposure	Duration of the exposure	Age at the start	Effect on brain	Effect on behavior	Reference
APPswe	Enriched housing (multiple mice in a large bin containing an inner cage with platforms, passageways, running wheels, toys, and novel habitats) + novel complex environment 3x weekly for several hours	4 months	16 months	No differences in total A*β* load	Improved MWM performance	[91]

TgCRND8	Enriched housing (equipped with diverse physically and cognitively stimulating objects, for example, gnawing wood, tunnels, balls, running wheels, and ladders)	4 months	1 month	↑ angiogenesis ↑ ApoE, LRP1, A2M ↓ RAGE	Not measured	[92]

TgCRND8	Enriched housing (equipped with diverse physically and cognitively stimulating objects, for example, gnawing wood, tunnels, balls, running wheels, and ladders)	4 months	1 month	↑ BrdU-positive cells ↑ synaptophysin immunoreactivity in the hippocampus	Not measured	[93]

TgCRND8	Enriched housing (plastic inset, wooden climbing frame, and a nesting material)	4 months	1 month	Not measured	↑ exploratory behavior ↓ anxiety-related behavior No effects in learning and memory, as assessed with MWM, barrier test, open-field, elevated plus maze, object recognition task, and Barnes maze	[94]

APP23	Enriched environment (multiple mice housed in large cages with a rearrangeable system of plastic tubes and cardboard boxes)	1 months	6, 18 months	No differences in plaque load ↑ DCX/CR ratio ↑ DCX- and calretinin-positive neurons in the hippocampus		[95]

APP23	Enriched housing (spacious cage equipped with a rearrangeable system of tubes, a cardboard box house, wire mesh ladders, and a crawling ball)	9 months	2 months	No differences in plaque load in neocortex or hippocampus ↑ hippocampal neurogenesis (DCX, calretinin) ↑ BDNF, NT-3 in the hippocampus	Improved learning and memory in MWM	[96]

APPswe/PS1ΔE9	Enriched housing (multiple mice in a large cage with crawl-tubes, platforms, running wheels and toys, changed weekly)	6 months	1.5 month	↓ total A*β* deposition, 28% in the hippocampus and 36% in entorhinal cortex ↑ synaptophysin in CA1 and CA3	Improved performance in MWM, RAWM	[97]
APPswe/PS1ΔE9	Enriched environment (large cages, running wheels, colored tunnels, toys, and chewable material)	1 month, 3 hours daily, next 4 months three times per week	1 month	↓ neocortical and hippocampal A*β* deposits ↑ increased neprilysin expression	Not measured	[98]

PS1/PDAPP	Enriched housing (multiple mice in a large bin containing an inner cage with platforms, passageways, running wheels, toys, and novel habitats) + novel complex environment 3/weekly for several hours	5 months	weaning	↑ gene expression of TTR, NF-*κ*B inhibitors,	Improved performance in MWM, RAWM and platform recognition tasks	[99]

APPswe/PS1ΔE9	Enriched housing (larger cages with running wheels, plastic play tubes, cardboard boxes, and nesting material that were changes or rearranged weekly)	6 months	2 months	↑ 68% of plaque area in the hippocampus ↑ 52% of total A*β* in hippocampus	Not measured	[100]

APPswe/PS1ΔE9	Enriched housing (larger cages with running wheels, plastic play tubes, cardboard boxes, and nesting material that were changes or rearranged weekly)	6 months	2 months	↑ 50% A*β*42 in the hippocampus ↑ 25% in hippocampal plaque load	Improved performance in MWM, RAWM	[101]

APOE3, APOE4	Cages with exploratory objects (toys, tunnels, and running wheels)	5 months	3 weeks	improvement in T-maze performance in APOE3 only	↑ expression of NGF ↑ Synaptophysin in the hippocampus of APOE3 only	[102]

APOE3, APOE4	Enriched housing ( cage with running wheel, labyrinth, bedding, house, chains, and wooden blocks)	5 months	3 weeks	↑ hippocampal A*β* deposits in the APOE4	Not measured	[103]

APOE3, APOE4	Wheel running	6 weeks	10 months	↑ BDNF in both ↑ TrkB, PAK and synaptophysin only in APOE4	Improved performance in place recognition in both genotypes Improved RAWM in APOE4 only	[104]

**Table 4:** Environmental exposure to metals and electromagnetic fields in transgenic mouse models of Alzheimer's disease.

Mouse model	Exposure	Duration of the exposure	Age at the start	Effect on brain	Effect on behavior	Reference
TgV337M	Aluminum-mltolate i.p. injection at various concentrations (50–100–200 *μ*M)	Max 14 days	3 months	Al levels were too low to induce changes in tau phosphorylation in brain homogenates, but Al concentration was lethal	Not measured	[146]

Tg2576	Dietary aluminum (2 mg/kg diet)	9 months	3 months	↑ soluble and insoluble A*β*40 and A*β*42 in neocortical and hippocampal homogenates ↑ plaque load in hippocampus and neocortex ↑ oxidative stress markers	Not measured	[115]

Tg2576	Dietary aluminum lactate (1 mg/g diet)	120 days	5 months	No significant differences in A*β*40 and A*β*42 in cortical homogenates, no alterations in proliferation, survival or differentiation of BrdU-positive neurons in DG	No improvement MWM	[117]

Tg2576	Dietary aluminum lactate (1 mg/g diet)	6 months	5 months	↑ Al concentration in hippocampus and cerebellum ↑ Cu in hippocampus ↓ Fe in cerebellum ↑ Mn and Zn in neocortex, hippocampus and cerebellum	Not measured	[118]

Tg2576	Dietary aluminum lactate (1 mg/g diet)	6 months	5 months	↑ Al concentrations in the hippocampus, but no difference between WT and Tg animals, no difference in oxidative stress reaction in the hippocampus between WT and Tg	Not measured	[116]

APPswe/PS1ΔE9	Iron carbonyl (1 mg/ml)	3 days	P12	No difference in A*β* plaque load in hippocampus and temporal cortex, no difference in microglial activity ↑ GFAP levels in temporal cortex ↑ saturated fatty acids ↓ unsaturated fatty acids ↓ oxidative damage markers	Not measured	[130]

APPswe/PS1ΔE9	Zinc-deficient (<10 parts Zn per million (ppm))	3 months	9 months	No significant difference in serum zinc levels, no difference cortical volume ↑ 25% in total plaque volume, no difference in number of plaques or laminar distribution, no difference in oxidative stress markers	Not measured	[136]

Tg2576	Zinc in drinking water (10 ppm/0.153 mM Zn)	±12 months	From conception	↓ A*β* deposits in hilar and molecular region of the DG	↓ spatial memory in MWM both in Tg and WT, buth most pronounced in Tg	[137]

TgCRND8	Zinc in drinking water (10 ppm/0.153 mM Zn)	5 months	From weaning	No significant differences	↓ spatial memory in MWM both in Tg and WT, buth most pronounced in Tg	[137]
TgC100	Zinc in diet (ZnSO_4_, 1000, 500 or 300 ppm)	15 months	7 weeks	↑ Brain Zn levels in brain homogenates ↓ Cu levels (n.s.) ↓ Cu/Zn ratio ↓ 13% soluble A*β* 40 (trend) No changes in GFAP, SOD1, APP, C100, nor NF200 (neuron loss), no difference in intensity or distribution of A*β* or GFAP staining	Not measured	[138]

TgC100	Copper in diet (CuSO_4_ 150 or 100 ppm)	7 weeks	9 months	No significant differences in Cu levels in brain homogenates ↑ Zn levels ↓ 18% soluble A*β* 40, no changes in GFAP, SOD1, APP, C100, nor NF200 (neuron loss), no difference in intensity or distribution of A*β* or GFAP staining	Not measured	[138]

3xTg	Copper sulfate (250 ppm) in 5% sucrose drinking water	3 or 9 months	2 months	*3 months exposure*: ↑ steady-state levels APP, C99 and BACE1 ↑ A*β* 40 in total plaque load in hippocampus, no alterations in total tau, phospho-tau nor Thy1.2 transcription activity ↑ AT8-positive neurons in CA1, no changes in steady-state levels of cdk5, p35/p25, GSK-3*β* or phospho-GSK-3*β* ↓ SOD1 activity in brain homogenates *9 months exposure: * ↑ steady-state levels APP, C99, C83, BACE1, ADAM10 ↑ soluble A*β*40, phospho-tau, no alterations total tau levels ↑ p25 formation ↓ SOD1 activity	Not measured	[143]

A*β*PPsw	Electromagnetic field exposure (918 MHz, 0.25 W/kg ± 2 dB) 2 × 1 h p/d	7-8 month exposure	2 months 5 months	*Young adult 7 months exposure, *no significant differences in soluble A*β* in hippocampus + neocortex, no effect op hippocampal DNA repair enzymes, antioxidant enzyme markers, protein oxidative damage, nor striatal DNA oxidation *Aged adult 8 months exposure * ↓ A*β* plaque load in hippocampus (−35%) and entorhinal cortex (−32%) ↑ soluble A*β* in hippocampus + neocortex	*Young adult 7 months exposure * Prevention of cognitive deficits in retroactive interference ↑ Y-maze spontaneous alternation level No differences in open field activity, balance beam, string agility, and elevated plus maze *Aged adult 8 months exposure * Reversal of cognitive deficits	[27]

**Table 5:** Traumatic brain injury in transgenic mouse models of Alzheimer's disease.

Mouse model	Exposure	Duration exposure	Age at start exposure	Effects on brain	Effects on behaviour	Reference
APP-YAC	Cortical contusion impact (3-mm diameter impounder onto the left parietal cortex, 100 ms; velocity (*v*) = 4.8−5.2 m/s; depth = 1 mm)	—	10–16 months	No difference	No difference in MWM	[149]

PDAPP	Cortical impact brain injury (3-mm diameter impounder onto the left parietal cortex, 100 ms; *v* = 4.8−5.2 m/s; depth = 1 mm)	—	4 months	Increased hippocampal neuronal death	MWM memory impairment in transgenic as compared to controls	[150]

PDAPP	Controlled cortical impact (3-mm diameter impounder onto the left parietal cortex, 100 ms; *v* = 4.8−5.2 m/s; depth = 1 mm)	—	6 months	Hippocampal atrophyDecrease the hippocampus and cingulate cortex 3 months after TBI	Not measured	[156]

PDAPP	Controlled cortical impact (3-mm diameter impounder onto the left parietal cortex, 100 ms; *v* = 4.8−5.2 m/s; depth = 1 mm)	—	24 months	Increased hippocampal neuronal loss and gliosis Regression of A*β* in the hippocampus	Not measured	[157]

APPswe	Controlled cortical impact (3-mm diameter impounder onto the left parietal cortex, 47 ms; *v* = 5.82 m/s; depth = 1.2 mm, driving pressure 73 psi)	—	3 months	2× increase in A*β*40 and A*β* 42 Reduced CA3 ynaptophysin immunoreactivity	MWM performance deficit	[152]

Tg2576	Controlled cortical impact (mild to moderate, 2-mm diameter impounder onto the right cortex, *v* = 3.3 m/s; depth = 1 mm)	—	22 months	Gene expression differences in inflammation, immune response and cell death	Not measured	[153]

Tg2576	Controlled cortical impact (3-mm diameter impounder onto the left parietal cortex, 100 ms; *v* = 4.8−5.2 m/s; depth = 1 mm)	Repetitive (2×)	9 months	Increased hippocampal amyloid deposition	MWM cognitive dysfunction	[154]

**Table 6:** Dietary, nutritional, and lifestyle habits and mouse models of Alzheimer's disease.

Mouse model	Exposure	Duration of the exposure	Age at the start	Effect on brain	Effect on behavior	Reference
APPswe/ind	Caloric restriction, 40%	2 weeks	3 months	↓ 40% in cortical and hippocampal plaque load	Not measured	[210]

APP_swe_/PS1_M146L_	Caloric restriction, 40%	4 months	2 months	↓ 55% in cortical plaque load	Not measured	[210]

3xTg	Caloric restriction, 40%	7 or 14 months	3 months	↓ hippocampal A*β*40, 42	Improved open field activity Improved performance in MWM	[212]

APP/PS1 KI	Western (40% fat) diet	1 month	1 month	↑ oxidative stress markers (protein nitrosylation, protein carbonyls, and lipid hydroperoxides)	Not measured	[167]

APPswe/PS1ΔE9	DHA	12 months	6 months	↓ A*β* deposition in the vasculature of the cingulate gyrus	Not measured	[168]

3xTg	Western (35% fat, low n-3 : n-6 PUFA ratio)	9 months	4 months	↑ cortical A*β*40, 42 ↑ cortical tau ↑ cortical GFAP	Not measured	[217]

Tg2576	High-cholesterol diet (5% cholesterol, 10% fat, 2% sodium cholate, and 5.2 kcal/g)	2 months	1 month	↑ soluble A*β*40, 42 ↑ plaque number and size in neocortex and hippocampus ↑*β*-CTFs	Not measured	[175]

3xTg	DHA-rich diet (1.3 g/100 g)	3, 6, 9 months	3 months	↓ soluble A*β*40, 42 ↓ PS1 levels ↓ somatodendric accumulation of tau ↓ tau phosphorylation (whole-brain homogenates)	Not measured	[179]

Tg2576	DHA-rich diet (0.6% DHA)	3 months	19 months	↓ 38% insoluble A*β* in cortex ↓ A*β*42 49% ↓ A*β*40 47.5% ↓ plaques by 39% in hippocampus, 49% in parietal cortex and 47% in perirhinal cortex	Not measured	[177]

APPswe/PS1ΔE9	Fish oil-based diet (0.4% DHA, 0.4% EPA, and 0.2% arachidonic acid)	4 months	6 months	↓ A*β*40, 42 levels in the hippocampus	Not measured	[178]

APPswe/PS1ΔE9	DHA rich diet (0.6% with safflower oil)	3 months	3 months	↓ plaque load by 27–30% in neocortex, ventral hippocampus, striatum, only in females ↑ hippocampal synaptotagmin and drebrin in females	Not measured	[180]

APPswe/PS1ΔE9	DHA rich diet (0.4%, low-saturated fatty acids, high PUFA, and low n-6/n-3 ratio)	6–13 months	2 months	↓ rCBV in 8 months and ↑ in 15 months in cerebral cortex	No diet effects on open field, MWM, reverse MWM, 12 circular hole board tasks	[184]
Tg2576	Folate, B6, and B12 deficiency	7 months	8 months	↑ A*β*40, 42 peptides in neocortex and hippocampus ↑ plaques in neocortex and hippocampus	Not measured	[186]

TgCRND8	Folate, B6, and B12 deficieny	3 months	1 month	↑ PP2A, GSK3b mRNA ↑ tau phosphorylation	Not measured	[188]

APPswe	Folic acid deficiency	3 months	7 months	20% loss of neurons in CA3 ↑ DNA damage in hippocampus, measured by DNA strand breaks	Not measured	[189]

Tg19959	Thiamine deficiency	10 days	2 months	↓ KGDHC (*α*-ketoglutarate dehydrogenase complex) ↑ neuronal loss in thalamus ↑ TNF-a and antimalondialdehyde, markers for oxidative stressAcceleration of amyloid plaques, ↑ total and insoluble A*β* ↑ BACE1 levels	Not measured	[190]

APP_swe_/PS1_Leu235Pro_	CoQ10 supplementation (2400 mg/kg/day, oral)	2 months	19 months	↓ hemisphere and hippocampal atrophy	Not measured	[36]

APPswe/PS1ΔE9	Retinoic acid (20 mg/kg, i.p.)	2 months	5 months	↓ A*β* plaque levels in frontal cortex and hippocampus ↓ APP-CTFs, 70% neocortex, 50% hippocampus, ↓ tau phosphorylation ↓ GFAP	Improved MWM performance	[191]

APP_swe_/PS1_M146L_	Caffeine (0.3 mg/ml in H_2_O)	1 month	19 months	↓ of total A*β*, 46% in entrorhinal cortex, 40% in the hippocampus ↓ soluble A*β*42, neocortex 51%, hippocampus 59% ↓ soluble A*β*40, neocortex 25%, hippocampus 37% ↓cRaf-1 in hippocampus	Improved working memory in RAWM	[196, 197, 218]

APP_swe_/PS1_M146L_	Caffeine (0.3 mg/ml in H_2_O)	4 months	4 months	↓ insoluble A*β*42 32%, soluble A*β*40 37% in the hippocampus ↓ expression of PS1 and BACE1	Improved performance in MWM, RAWM, platform recognition	[218]

APP_swe_/PS1_M146L_	Caffeine (1.5 mg/0.2 ml, oral)	2 months	15–20 months	↓ soluble A*β* 42 in neocortex and hippocampus, by 51% and 59%, respectively	Improved RAWM performance	[197]

Tg2576	Green tea derived EGCG (50 mg/kg, oral in H_2_O)	6 months	8 months	↓ 50% in plaque load in hippocampus, cingulate and entorhinal cortex	Improved performance in RAWM	[199, 200]

Tg2576	EGCG (20 mg/kg,i.p.)	2 months	12 months	↓ 50 % in soluble A*β*40, 42 ↑*α*-secretase cleavage by 40%	Not measured	[200]

Tg2576	Luteolin (20 mg/kg, i.p.)	1 month	8 months	↓ GSK3 activity ↓ PS1 processing	Not measured	[201]

Tg2576	Cabernet sauvignon wine (6% final ethanol concentration in H_2_O)	7 months	4 months	↓ A*β*40, 42 peptides in neocortex and hippocampus ↑ CTF cleaved fragment in neocortex ↑*α*-secretase activity	Improved performance in Barnes maze task	[202]

3xTg	Nicotine (0.025–0.6 mg/ml, in H_2_O)	5 months	1 months	↑ hippocampal A*β*42 AChRs ↑ tau phosphorylation in the hippocampus ↑ p38-MAP kinase	Not measured	[209]

Tg2576	Nicotine (0.25–45 mg/kg, in H_2_O)	10 days	9 months	↓ soluble A*β*40, 42 in neocortex	Not measured	[207, 208]

Tg2576	Nicotine (25–35 mg/kg, in H_2_O)	5.5 months	9 months	↓ A*β*42 plaques in the neocortex, hippocampus, and olfactory bulb	Not measured	[207]

APPswe	Curcumin (160–5000 ppm)	6 months	10 months	↓ oxidized proteins in neocortex and hippocampus ↓ soluble A*β* in neocortex and hippocampus ↓ total area and number of plaques	Not measured	[214]

Tg2576	*Ginko biloba* (0.35 mg/kg, in H_2_O)	6 months	8 months	No differences in plaque load	Improved performance in MWM	[216]
